# EGCG Reduces Obesity and White Adipose Tissue Gain Partly Through AMPK Activation in Mice

**DOI:** 10.3389/fphar.2018.01366

**Published:** 2018-11-22

**Authors:** Fang Li, Chen Gao, Ping Yan, Meng Zhang, Yinghao Wang, Yue Hu, Xiaoyun Wu, Xuanjun Wang, Jun Sheng

**Affiliations:** ^1^Key Laboratory of Puer Tea Science, Ministry of Education, Yunnan Agricultural University, Kunming, China; ^2^Research Center for Tea Processing of Yunnan, Yunnan Agricultural University, Kunming, China; ^3^Scientific Observing and Experimental Station of Tea Resource and Processing in Yunnan, Ministry of Agricultural, Kunming, China; ^4^Department of Science, Yunnan Agricultural University, Kunming, China

**Keywords:** EGCG, obesity, white adipose tissues, gene expression, AMPK

## Abstract

(-)-Epigallocatechin-3-gallate (EGCG), which is the most abundant catechin in green tea, has many potential health benefits, including decreased weight gain and/or adipose tissue weight. Suggested mechanisms for body weight reduction by EGCG include: (1) a decrease in calorie intake and (2) activation of AMPK in liver, skeletal muscle, and white adipose tissue. However, only one study supports the AMPK hypothesis. To determine the role of AMPK in EGCG-induced reduction of body weight, we administrated 50 mg/kg and 100 mg/kg per day to mice, together with a high-fat diet (HFD), for 20 weeks. EGCG had a significant effect on obesity and decrease in epididymal adipose tissue weight, and also affected serum lipid characteristics, including triglyceride, cholesterol (CHOL), and high- and low-density lipoprotein CHOL (HDL-C, LDL-C) concentrations. In addition, EGCG increased the excretion of free fatty acids from feces. By measuring the mRNA expression levels of genes involved in lipid metabolism, we found that EGCG inhibited the expression of genes involved in the synthesis of *de novo* fatty acids (*acc1*, *fas*, *scd1*, *c/ebp*β, *ppar*γ, and *srebp1*) and increased the expression of genes associated with lipolysis (*hsl*) and lipid oxidization in white adipose tissue, in both the HFD and the EGCG groups. However, EGCG significantly increased the expression of genes involved in the synthesis of *de novo* fatty acids compared with the HFD group. Increased AMPK activity was found in both subcutaneous and epididymal adipose tissues. In conclusion, EGCG can decrease obesity and epididymal white adipose tissue weight in mice, only partially *via* activation of AMPK.

## Introduction

Overweight and obesity have increased at alarming rates in recent years and are now prevalent throughout the world. Overweight and obesity are usually caused by an energy imbalance between calorie intake and consumption, in particular, a high dietary intake of fats and sugars (Bolton-Smith and Woodward, 1994; Gibson, 1996; Yeomans, 2017; Zambrano and Nathanielsz, 2017). A population-based study from 1975 to 2014, with more than 19.2 million adult participants, revealed that about 641 million individuals worldwide were obese in 2014, compared with 150 million in 1975. Over the past four decades, a global transition has taken place, from a world in which the prevalence of underweight was more than double that of obesity, to one in which more people are obese than underweight (NCD Risk Factor Collaboration [NCD-RisC] et al., 2016). There is a strong positive association between obesity and various chronic diseases, including type 2 diabetes, high blood pressure, cardiovascular disease, stroke, and even some cancers (Rossner, 2002; Basen-Engquist and Chang, 2011; Sartorius et al., 2016; Smith et al., 2017). These are harmful to the individual’s health, as well as creating a great economic burden for their families. Therefore, effective weight management interventions, including low-calorie diets and exercise, might have an important role in alleviating obesity and metabolic diseases (Lyznicki et al., 2001).

Green tea is consumed worldwide, especially in East Asia. The main components of green tea are caffeine and polyphenolic compounds known as catechins. Of the catechins, (-)-epigallocatechin-3-gallate (EGCG) is the most abundant in green tea. EGCG has been reported to be responsible for many of the potential health benefits of green tea, including decreased weight gain and alleviated insulin resistance (Richard et al., 2009; Sae-tan et al., 2011).

In recent years, some studies have shown that the consumption of green tea extract or EGCG could significantly reduce gain of body weight and/or adipose tissue, decrease blood glucose or insulin levels, and increase insulin sensitivity or glucose tolerance in rodents, compared with those fed high-fat diets (HFDs) or genetically obese/diabetic animal models. Bose et al. found that supplementation with dietary EGCG (3.2 g/kg diet) for 16 weeks significantly reduced body weight gain, body fat percentage, and visceral fat weight in mice compared with those not receiving EGCG treatment (Bose et al., 2008). Lee et al. fed an HFD to C57BL/6J mice for 8 weeks to induce obesity; subsequently, the mice were divided into three groups and maintained on a high-fat control diet or on an HFD supplemented with 0.2 or 0.5% EGCG (w/w) for further 8 weeks. EGCG significantly reduced the body weight and mass of various adipose tissues, and considerably reduced levels of plasma triglycerides (TAGs) and liver lipids (Lee et al., 2009). Chen et al. also found that EGCG treatment significantly reduced body weight gain in mice with high-fat/Western-style diet (HFW)-induced obesity, attenuated insulin resistance, and decreased blood glucose and liver TAG levels compared with the HFW-only group (Chen et al., 2011). Ortsater et al. found that dietary supplementation of EGCG (1% of diet) significantly reduced body weight and also prevented the progression of glucose intolerance in *db/db* mice (Ortsater et al., 2012). Several epidemiological studies revealed the beneficial effects of tea and EGCG on obesity in humans; however, the results were not always positive in human participant studies (Suzuki et al., 2016). For example, Baladia et al. performed a systematic review and meta-analysis, and found that green tea extracts had no statistically significant effect on the weight of overweight or obese adults; there was a small effect on the decrease in the percentage of fat mass (Baladia et al., 2014). Therefore, loss of body fat may require a higher intake of EGCG or other catechins, or the addition of metabolic stimulants (Yang et al., 2016).

Yang et al. (2016) reviewed the mechanisms of body weight reduction and alleviation of metabolic syndrome by green tea. They proposed two major mechanisms involving EGCG: (1) decreased absorption of lipids and proteins in the intestine, thus reducing calorie intake and (2) activation of AMPK in liver, skeletal muscle, and white adipose tissue. According to their “AMPK hypothesis,” AMPK plays a major part in mediating the actions of EGCG on fatty acid synthesis and fatty acid catabolism. However, only one study has reported activation of AMPK by EGCG in cultured cells and in liver tissues of mice, suggesting that the effects of catechins, including their anti-obesity and anti-cancer effects, are at least partially mediated by activation of AMPK (Murase et al., 2009). Thus, it is still unknown whether activation of AMPK has a central role in the reduction of adipose tissue by EGCG, and whether AMPK has similar roles in different depots.

In this study, we administered different doses of EGCG to C57BL/6J mice to determine its anti-obesity and hypolipidemic effects. We also aimed to explore (1) whether EGCG-activated AMPK phosphorylation played a central part in reducing the accumulation of fats in adipose tissues and (2) whether EGCG had similar effects on the regulation of lipid accumulation in different adipose depots.

## Materials and Methods

### Animals

Male C57BL/6J mice around 6 weeks old were obtained from Changzhou Card Vince Experimental Animal Co., Ltd., C57BL/6J mice were housed individually in an environmentally controlled room (ventilation, 22 ± 2°C, 55 ± 5% relative humidity) with *ad libitum* access to water and food, and maintained on a 12-h light–dark cycle. EGCG (purity > 98%, Chengdu Biopurify Phytochemicals Ltd.) was prepared with distilled water as a 10 mg/mL storage solution and stored in the dark at -20°C until use for gavage.

Mice were fed with a control diet containing 10 kca% fat (D12450J, Research Diets) 2 weeks prior to initiating the experiment. The mice were divided into four groups at around 8 weeks old; each group included eight male mice. Mice in the control group were fed a control diet (D12450J, Research Diets). Mice in the HFD group were fed a high-fat diet containing 60 kca% fat (D12492, Research Diets). Mice in the HFD + EGCG groups were fed the HFD and, separately, 50 mg/kg of EGCG and 100 mg/kg EGCG were administered intragastrically per day. After 20 weeks of feeding, changes in body weight, food intake, water intake, and blood glucose were recorded. The mice were then sacrificed and tissues were collected, immediately frozen with liquid nitrogen, and stored at -80°C. To estimate the obesity of mice in this study, we used the Lee index [body weight (g)^∗^0.33^∗^1,000/body length (cm)], a widely used method to judge body weight and obesity in small-sized experimental animals (Lee, 1929; Bernardis and Patterson, 1968). The animal experiments in this study were approved by the animal ethics committee of Yunnan Agricultural University (No 20160318002, March 18, 2016).

### Real-Time Quantitative Polymerase Chain Reaction (PCR) Analysis

Total RNA from adipose tissues was extracted using TRIzol (TransGen Biotech, Beijing, China). Complementary DNA was synthesized using the PrimeScript RT reagent kit with genomic DNA (Takara) according to the manufacturer’s protocol. PCR was performed and quantified using SYBR Green real-time PCR Master Mix (TransStart Top Green qPCR SuperMix, TransGen Biotech, Beijing, China) with an ABI 7900HT Fast Real-Time PCR System (Applied Biosystems, Inc.). Data were obtained after PCR cycling; gene expression levels were calculated by the 2^-ΔΔCT^ method and normalized to β-actin measured in parallel. The primer sequences are provided in Table 1.

**Table 1:** qRT-PCR primers used in this study.

Gene name	Forward primer (5′-3′)	Reverse primer (5′-3′)
*ppar*γ	GTGCCAGTTTCGATCCGTAGA	GGCCAGCATCGTGTAGATGA
*c/ebp*β	ACAAGGCCAAGATGCGCAAC	TTCCGCAGGGTGCTGAGCT
*ap2*	AATCACCGCAGACGACAG	ACGCCTTTCATAACACATTCC
*atgl*	TGTGGCCTCATTCCTCCTAC	TCGTGGATGTTGGTGGAGCT
*pgc1*α	CCCTGCCATTGTTAAGACC	TGCTGCTGTTCCTGTTTTC
*fas*	GGAGGTGGTGATAGCCGGTAT	TGGGTAATCCATAGAGCCCAG
*acc1*	CGCTCGTCAGGTTCTTATTG	TTTCTGCAGGTTCTCAATGC
*scd1*	GCTGGAGTACGTCTGGAGGAA	TCCCGAAGAGGCAGGTGTAG
*srebp1c*	GGAGCCATGGATTGCACATT	GGCCCGGGAAGTCACTGT
*hsl*	TTCTCCAAAGCACCTAGCCAA	TGTGGAAAACTAAGGGCTTGTTG
*ppar*α	AGGAAGCCGTTCTGTGACAT	TTGAAGGAGCTTTGGGAAGA
*aco2*	GGTGGTGATTGGAGATGA	CTTTAGATTGGTTTCGTGGA
*ucp2*	ATGGTTGGTTTCAAGGCCACA	CGGTATCCAGAGGGAAAGTGAT
*mcad*	CCTAAGGCTCCTGCCAGTAAA	AACCAGCTCCCTCACCAAGTAA
*β-actin*	GAGACCTTCAACACCCCAGC	ATGTCACGCACGATTTCCC

### Western Blot Analyses

Total proteins were isolated from white adipose tissues using RIPA (Solarbio Life Science, Beijing, China) buffer supplemented with protease and phosphatase inhibitors. Protein concentrations were determined using a protein assay reagent (Beyotime Biotechnology, China) and equal amounts of protein (60 μg) were loaded in each well of an 8% sodium dodecyl sulfate polyacrylamide gel electrophoresis gel. After the proteins were transferred onto a polyvinylidene difluoride membranes, the blots were blocked with 5% bovine serum albumin, followed by incubation overnight at 4°C with the following primary antibodies: anti-AMPKα (23A3), anti-phospho-AMPKα (Thr172), anti-ACC, anti-FAS, and anti-CPT1α (Cell Signaling Technology, United States). After three washes with TBST buffer, the blots were incubated with appropriately diluted horseradish peroxidase-conjugated secondary antibodies for 1 h at room temperature. The immunoblots were visualized with an enhanced chemiluminescence detection kit (Tiangen Biotech, Beijing, China). Protein levels were normalized to tubulin as a loading control.

### Detection of Serum Lipids and Fecal Lipids

Blood was taken from the inside canthus and collected in micro-centrifuge tubes with heparin sodium. The blood samples were centrifuged at 3,000 rpm for 20 min, and the plasma was kept frozen at -20°C until analysis. Total cholesterol (TC), TAG, high-density lipoprotein cholesterol (HDL-C), and LDL-C levels were measured. All blood sample analysis took place at the Second Affiliated Hospital in Kunming.

Fecal samples were collected at 18 weeks, frozen at -80°C, and freeze-dried (FD5-6, Sim). Levels of TC, TAG, HDL-C, LDL-C, and free fatty acids (FFAs) were measured using the relevant kits (Nanjing Jiancheng Bioengineering Institute, Nanjing, China) according to the protocol.

### Statistical Analysis

Statistical analysis of the experimental data was performed using SPSS 17.0 and GraphPad Prism 6. Data are presented as mean ± SEM. Differences between groups were analyzed using one-way ANOVA; *P* < 0.05 was considered significant.

## Results

### EGCG Reduced Adipose Tissue Weight Gain

**FIGURE 1:**
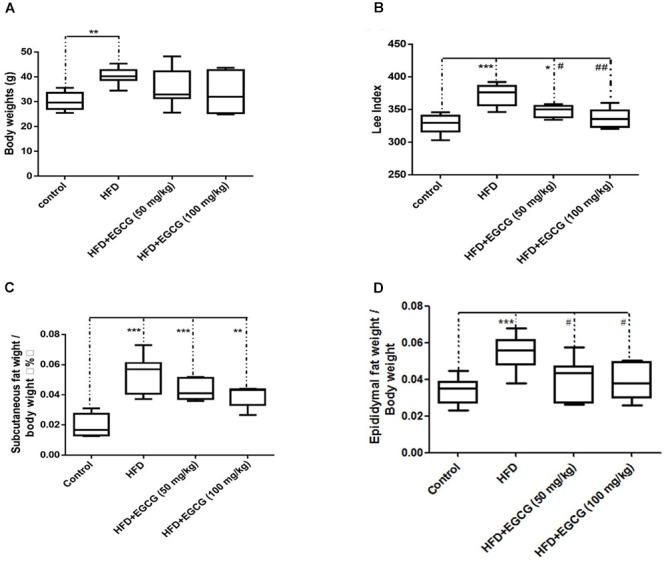
EGCG reduced obesity and the adipose weight in mice. **(A)** The body weights of mice after 20 weeks feeding; **(B)** Lee index; **(C)** ratio of subcutaneous fat weight to body weight; and **(D)** epididymal fat weight to body weight. Data were shown as mean ± SEM (*n* = 6/7 each group). ^∗^*P* < 0.05; ^∗∗^*P* < 0.01; ^∗∗∗^*P* < 0.001 vs. control. ^#^*P* < 0.05; ^##^*P* < 0.01; ^###^*P* < 0.001 vs. HFD.

The body weights of mice fed the HFD increased significantly than those of control mice; however, administration of EGCG did not reduce gain of body weight compared with control and HFD mice (Figure 1A). Then we determined the obesity of mice using the Lee index [body weight (g)^∗^0.33^∗^1,000/ body length (cm)]. We found that the Lee index was significantly higher in HFD mice than mice in other groups, and also EGCG significantly reduced obesity in mice (Figure 1D). We also determined the ratios of subcutaneous fat weight to body weight (SFW/BW) and epididymal fat weight to body weight (EFW/BW) (Figures 1C,D). SFW/BWs in the HFD and EGCG groups were all significantly higher than those in the control group; however, administration of EGCG did not significantly reduce gain of subcutaneous fat compared with the HFD group (Figure 1C). The EFW/BWs in the HFD group were significantly higher than those in the other groups (Figure 1D). These results indicated that administration of EGCG could reduce obesity and the accumulation of epididymal fat in mice.

### EGCG Changed Serum Lipid and Fecal Lipid Characteristics

**FIGURE 2:**
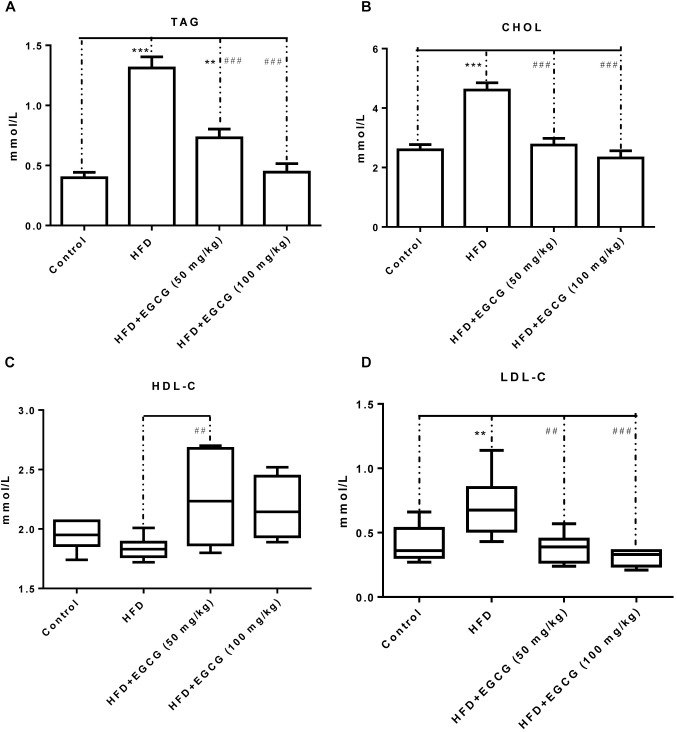
EGCG alleviated HFD-induced hyperlipidemia in mice. **(A)** TAG; **(B)** CHOL; **(C)** HDL-C; and **(D)** LDL-C. Data were shown as mean ± SEM (*n* = 6/7 each group). ^∗^*P* < 0.05; ^∗∗^*P* < 0.01; ^∗∗∗^*P* < 0.001 vs. control. ^#^*P* < 0.05; ^##^*P* < 0.01; ^###^*P* < 0.001 vs. HFD. ^§^ *P* < 0.05 vs. HFD + EGCG (50 mg/kg).

Concentrations of TAG, CHOL, and LDL-C were all significantly increased in the HFD group compared with the other groups, and the concentration of HDL-C was decreased in the HFD group (Figure 2). Administration of EGCG (50 mg/kg⋅ per day, 100 mg/kg⋅ per day) significantly decreased the concentrations of TAG, CHOL, and LDL-C induced by HFD (Figures 2A,B,D), and increased the concentration of HDL-C (Figure 2C).

**FIGURE 3:**
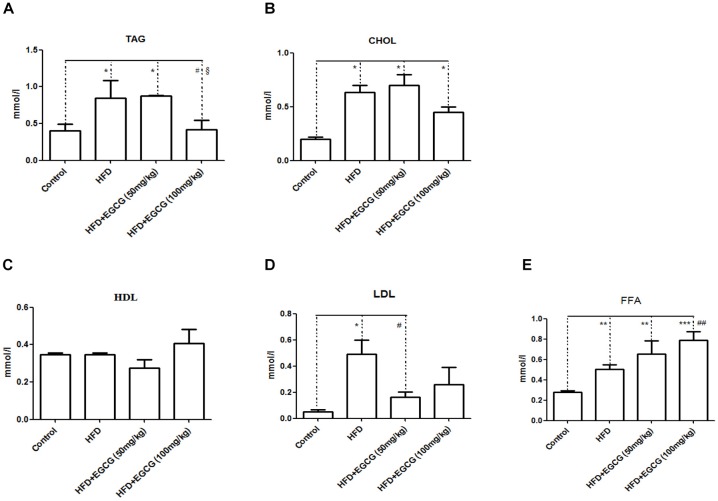
Effect of EGCG on lipid excretion. **(A)** TAG; **(B)** CHOL; **(C)** HDL-C; **(D)** LDL-C; and **(E)** FFA. Data were shown as mean ± SEM (*n* = 6/7 each group). ^∗^*P* < 0.05; ^∗∗^*P* < 0.01; ^∗∗∗^*P* < 0.001 vs control. ^#^*P* < 0.05; ^##^*P* < 0.01; ^###^*P* < 0.001 vs HFD. ^§^ *P* < 0.05 vs HFD + EGCG (50 mg/kg).

EGCG has previously been shown to increase fecal lipid content, suggesting decreased digestion and absorption of lipids (Bose et al., 2008; Friedrich et al., 2012). In this study, we found that fecal TAG, CHOL, and LDL excretion in the HFD group increased significantly than in the control group (Figures 3A,B,D). Administration of 50 mg/kg⋅per day EGCG with HFDs could also increase the excretion of fecal TAG and CHOL; however, administration of 100 mg/kg⋅per day EGCG with HFDs did not increase the excretion of fecal TAG and CHOL (Figures 3A,B). Also, there were no significant changes in concentrations of fecal HDL or LDL in the EGCG-treated groups compared with the control group (Figures 3C,D). We also found that the fecal excretion of FFA increased significantly with increasing dose of EGCG per day (Figure 3E).

### EGCG Modulated Lipid Metabolism Partly Through AMPK in White Adipose Tissue

**FIGURE 4:**
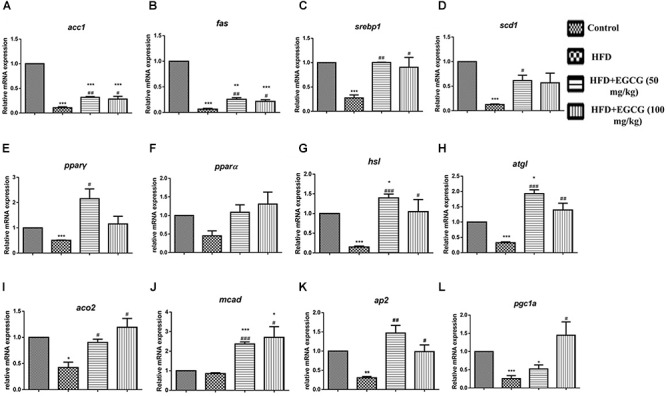
Effect of EGCG on the expression of genes involved in lipogenesis, oxidation, and transportation in subcutaneous adipose tissues of mice. Total RNA extracted from subcutaneous white adipose tissue was used for mRNA expression analysis of genes involved in fatty acid synthesis [**(A–E)** *acc1*, *fas*, *srebp1*, *scd1*, and *ppar*γ], lipolysis [**(F–J)** *ppar*α, *hsl*, *atgl*, *aco2*, and *mcad*], lipid transportation and thermogenesis [**(K–L)** *ap2*, *pgc1*α]. Values are the mean ± SEM (*n* = 3/4 each group). ^∗^*P* < 0.05; ^∗∗^*P* < 0.01; ^∗∗∗^*P* < 0.001 vs. control. ^#^*P* < 0.05; ^##^*P* < 0.01; ^###^*P* < 0.001 vs. HFD.

To explore the mechanisms by which EGCG reduced adipose indices in HFD mice, we separately determined the mRNA expression levels of genes associated with lipid metabolism in subcutaneous adipose tissue and epididymal adipose tissue. In subcutaneous adipose tissues, the expression of several genes for lipid synthesis, such as *acc1* and *fas*, decreased significantly in the HFD group; however, EGCG administration increased their expression significantly by comparison (Figures 4A,B, 6A). The relative expression levels of *ppar*γ, *srebp1*, and *scd1* were significantly increased in EGCG groups, although HFD significantly inhibited their expression (Figures 4C–E). We also found significant decreases in the relative mRNA expression of genes involved in lipolysis (*hsl*, *atgl*) and fatty acid oxidation (*ppar*α, *aco2*, *mcad*) in HFD mice; however, administration of EGCG could increase their expression to levels similar to those in the control group (Figures 4F–J). Expression levels of *ap2* and *pgc1*α, which are associated with fatty acid transfer and thermogenesis, were also significantly increased in EGCG-treated groups in subcutaneous fat tissues even when the mice were fed an HFD (Figures 4K,L).

**FIGURE 5:**
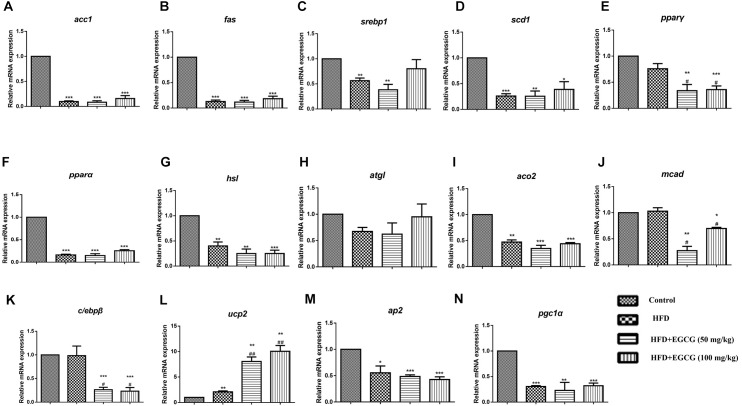
Effect of EGCG on the expression of genes involved in lipogenesis, oxidation, and transportation in epididymal adipose tissues of mice. Total RNA extracted from subcutaneous white adipose tissue was used for mRNA expression analysis of genes involved in fatty acid synthesis [**(A–E)** *acc1*, *fas*, *srebp1*, *scd1*, and *ppar*γ], lipolysis [**(F–J)** *ppar*α, *hsl*, *atgl*, *aco2*, and *mcad*], adipocypte differentiation transportation, and thermogenesis [**(K–N)** *c/ebp*β, *ap2*, *ucp2*, and *pgc1*α]. Values are the mean ± SEM (*n* = 3–4). ^∗^*P* < 0.05; ^∗∗^*P* < 0.01; ^∗∗∗^*P* < 0.001 vs. control. ^#^*P* < 0.05; ^##^*P* < 0.01; ^###^*P* < 0.001 vs. HFD.

**FIGURE 6:**
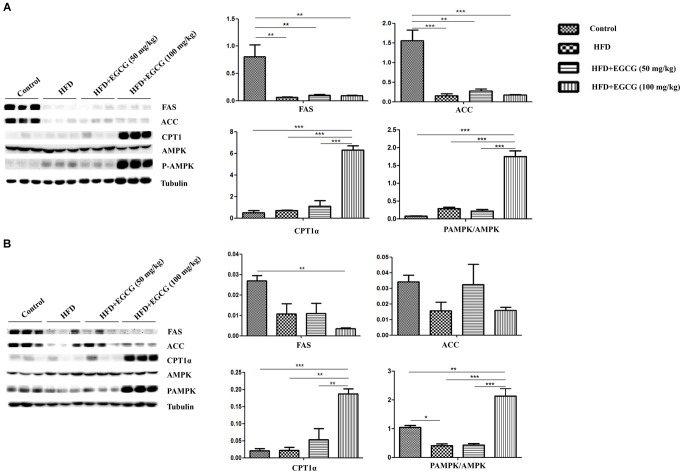
Effect of EGCG on the expression of proteins involved in lipogenesis, oxidation, and activation of AMPK. Lysate was prepared from subcutaneous adipose tissues and epididymal adipose tissues and subjected to Western blotting analysis to detect the expression of proteins involved in lipogenesis (FAS and ACC), oxidation (CPT1α), and activation of AMPK in **(A)** subcutaneous adipose tissues and **(B)** epididymal adipose tissues, separately. Values are the mean ± SEM (*n* = 3). ^∗∗^*P* < 0.01; ^∗∗∗^*P* < 0.001 between groups.

In epididymal adipose tissue, we also found that the expression of several genes for lipid synthesis, including *acc1*, *fas*, *scd1*, *c/ebp*β, *ppar*γ, and *srebp1*, decreased significantly in EGCG-treated mice; the protein expression level of FASN was only decreased significantly in the 100 mg/kg EGCG group, but that of ACC1 was not decreased in the HFD or EGCG groups (Figures 5A–F, 6B). The relative mRNA expression of the *hsl* gene, which is involved in lipolysis, was decreased significantly in HFD and EGCG mice (Figure 5G); however, the expression level of another gene associated with lipolysis, *atgl*, was similar among all the groups (Figure 5H). The relative mRNA expression levels of fatty acid oxidation genes (*ppar*α, *aco2*, and *mcad*) in EGCG mice compared with the control group, as well as the protein expression level of CPT1α was significantly decreased in 100 mg/kg EGCG mice compared with other groups (Figures 5I–K, 6B). The expression levels of *ap2*, *ucp2*, and *pgc1*α, which are associated with fatty acid transfer and thermogenesis, were also significantly reduced in EGCG-treated groups when mice were fed an HFD (Figures 5L–N).

Yang et al. has proposed that AMPK has a major role in mediating the effects of EGCG on fatty acid synthesis and fatty acid catabolism (Yang et al., 2016). Therefore, we determined the activity of AMPK. In both subcutaneous adipose tissues and epididymal adipose tissues, AMPK activity was significantly activated in 100mg/kg⋅d EGCG groups compared with other groups (Figures 6A,B).

## Discussion

The objective of the present study was to determine whether the fat-loss functions of EGCG involved similar effects on regulation of lipid accumulation in different adipose depots, as well as to explore the underlying mechanisms. In this study, we found that administration of EGCG, no matter 50 mg/kg per day or 100 mg/kg per day could not reduce gain of body weight (Figure 1A). However, EGCG had significant positive effects on obesity and epididymal fat accumulation in mice (Figures 1B,C,D). These results seemed contradictory in this study. The reason might be that our experiment had lasted for 20 weeks, some mice had higher body lengths along with higher body weights but not obese, which might have some effects on data analysis. So, we calculated the obesity difference among groups using Lee index, and found that EGCG could reduce obesity in mice (Figure 1B). Then we calculate the fat index of mice, and found that EGCG significantly reduced fat accumulation in epididymal fat tissues, but not in subcutaneous fat tissues. These results indicate that EGCG reduce obesity might mainly *via* reducing lipid accumulation in epididymal fat tissue. Our results were partially consistent to previous studies of Lee et al. (2009) and Chen et al. (2011). The reason might be that the doses of EGCG were different between our study and previous studies.

A combined transcriptomics and lipidomics analysis on different adipose tissues demonstrated that gene regulation in response to HFD and fatty acid patterns differed markedly between adipose depots (Caesar et al., 2010). A previous study by Caesar et al. also found that the average cell area in epididymal adipose tissues was larger than that of adipocytes in subcutaneous and mesenteric adipose depots after 12 weeks HFD feeding in male transgenic ApoE3 Leiden mice (Caesar et al., 2010). To explore whether EGCG had different effects on fat accumulation in subcutaneous and epididymal adipose tissues, we detected the expression of genes involved in the synthesis of *de novo* fatty acids and oxidization of fatty acids. We found that EGCG inhibited the expression of genes involved in the synthesis of *de novo* fatty acids (*acc1*, *fas*, *scd1*, *c/ebp*β, *ppar*γ, and *srebp1*) and increased the expression of genes associated with lipolysis (*hsl*) and lipid oxidization (*ppar*α, *aco2*, and *mcad*) in epididymal adipose tissues (Figures 5, 6B). Although EGCG accelerated lipolysis and fatty acid oxidation in subcutaneous adipose tissues, some adipogenic genes (*acc1*, *fas*, *scd1*, *ppar*γ, and *srebp1*) were significantly upregulated at the mRNA level by EGCG compared with the HFD group (Figure 4), but not at the protein level (Figure 6A). These results show that *de novo* lipogenesis was stably reversed by EGCG, whereas EGCG highly enhanced the mRNA level of delta-9 desaturase, which converts saturated fatty acids to monounsaturated fatty acids. It also appeared that fatty acid synthesis and desaturation were not co-regulated under the control of EGCG in subcutaneous adipose tissues. Our results suggest that EGCG might have different roles in lipogenesis in subcutaneous and epididymal adipose tissues. Therefore, EGCG might act *via* different mechanisms in subcutaneous and epididymal tissues, owing to the different gene regulation of these tissues in response to HFD. Yang et al. proposed the “AMPK hypothesis” to highlight the central role of AMPK in the regulation of genes involved in adipogenesis, lipogenesis, and lipolysis (Yang et al., 2016). Therefore, we examined the activity of AMPK in subcutaneous and epididymal adipose tissues. In both subcutaneous adipose tissue and epididymal adipose tissue, AMPK activity increased in the 100 mg/kg EGCG group compared with other groups (Figures 6A,B). Although AMPK was activated by EGCG in both subcutaneous adipose tissue and epididymal adipose tissue, this might only represent part of the process of EGCG-regulated lipid metabolism. It has previously been reported that activation of AMPK inhibits activation of ACC, FASN, SREBP1, and PPAR**γ (Long and Zierath, 2006; Hardie et al., 2012, Hardie, 2015; Wang et al., 2018). In this study, EGCG upregulated the expression of some adipogenic genes (*acc1*, *fas*, *scd1*, *ppar*γ, and *srebp1*) at the mRNA level but not at the protein level in subcutaneous adipose tissues (Figures 4A–E); in particular, it reduced the expression levels of *srebp1* and *scd1* to those observed in control mice (Figures 4C,D). These results indicated that expression of the lipogenic genes was not regulated by activation of AMPK. Therefore, our results suggest that there may exist other regulatory mechanisms of EGCG in adipose tissues; this requires further studies for clarification. Overall, our results indicate that EGCG might regulate lipid metabolism partly through AMPK in adipose tissues in mice. These results were partially consistent with a previous report by Murase et al. (2009), which also found that the anti-obesity and anti-cancer effects of EGCG were mediated, at least in part, by the activation of AMPK in various tissues.

In summary, we found that a higher dose of EGCG administrated to C57BL/6J mice moderately decreased body weights and adipose tissue indices in both subcutaneous and epididymal adipose tissues. Gene expression analysis of lipid metabolism indicated that EGCG exerted its effects *via* different mechanisms in subcutaneous and epididymal tissues, owing to their different gene regulation in response to HFD. Furthermore, AMPK appeared to have a minor role in the regulation of genes involved in the processes of adipogenesis, lipogenesis, and lipolysis in both subcutaneous and epididymal adipose tissues.

## Author Contributions

FL, CG, and PY performed the animal experiments, qPCR, Western blot, and data analysis. MZ helped in the animal experiments and some data analysis. YW and YH helped the qPCR. XuW, XiW, and JS designed the study. XiW wrote the manuscript.

## Conflict of Interest Statement

The authors declare that the research was conducted in the absence of any commercial or financial relationships that could be construed as a potential conflict of interest.
